# Thrombospondin‐4 Regulates Lipopolysaccharide‐Induced Apoptosis and Inflammation in Nucleus Pulposus Cells via the Phosphatidylinositol 3‐Kinase/Protein Kinase B Pathway

**DOI:** 10.1002/iid3.70484

**Published:** 2026-07-06

**Authors:** Yang Shu, Chunhua Sun, Shi Chen

**Affiliations:** ^1^ Department of Orthopedics The Sixth Hospital of Wuhan, Affiliated Hospital of Jianghan University Wuhan China; ^2^ Department of Hemodialysis Wuhan Fourth Hospital Wuhan China; ^3^ Department of Pulmonary and Critical Care Medicine Affiliated Hospital of Jianghan University Wuhan China

**Keywords:** apoptosis, inflammatory response, intervertebral disc degeneration, nucleus pulposus cells, PI3K/AKT pathway, THBS4

## Abstract

**Objective:**

This study investigated the expression profile of Thrombospondin‐4 (THBS4) in intervertebral disc degeneration (IDD) and clarify its regulatory role in lipopolysaccharide (LPS)‐induced apoptosis and inflammation in nucleus pulposus cells (NPCs) via the phosphatidylinositol 3‐kinase (PI3K)/protein kinase B (AKT) pathway.

**Methods:**

IDD‐related differentially expressed genes were screened through the integration of GeneCards and the Gene Expression Omnibus (GEO) database (GSE186542), followed by Kyoto Encyclopedia of Genes and Genomes (KEGG) pathway enrichment analysis to explore potential signaling mechanisms. An in vitro inflammatory injury model was established using LPS‐stimulated NPCs. The effects of THBS4 overexpression on cell proliferation, apoptosis, inflammatory cytokine secretion, and extracellular matrix protein expression were evaluated using reverse transcription‐quantitative polymerase chain reaction (RT‐qPCR), western blotting, 5‐ethynyl‐2′‐deoxyuridine (EdU) assay, flow cytometry, and enzyme‐linked immunosorbent assay (ELISA). Additionally, the involvement of the PI3K/AKT pathway in mediating THBS4‐related effects was confirmed using the PI3K inhibitor LY294002.

**Results:**

Bioinformatics analysis revealed that THBS4 was significantly downregulated in IDD and was closely linked to the PI3K/AKT pathway. Functional assays demonstrated that overexpression of THBS4 markedly enhanced NPCs proliferation, suppressed apoptosis, reduced the secretion of tumour necrosis factor alpha (TNF‐α), interleukin‐1beta (IL‐1β) and IL‐6, and increased the expression of IL‐10, Aggrecan, and Collagen type II. These protective effects were accompanied by activation of the PI3K/AKT pathway and were significantly reversed by LY294002 treatment.

**Conclusion:**

THBS4 alleviated LPS‐induced damage in NPCs through the activation of the PI3K/AKT pathway, exerting anti‐apoptotic and anti‐inflammatory effects; the specific upstream molecular mechanism of PI3K/AKT activation by THBS4 requires further investigation. These findings suggested that THBS4 may serve as a potential therapeutic target for IDD treatment.

## 1 Introduction

Intervertebral disc degeneration (IDD) is a major cause of chronic lower back pain with a steadily rising prevalence, posing a significant global health challenge that compromises work capacity and quality of life [1, 2]. Currently, there are no effective clinical interventions to reverse IDD progression, primarily because of the lack of evident early symptoms and an incomplete understanding of its molecular pathogenesis [3]. Therefore, identifying key regulatory factors is crucial for early intervention and the development of targeted molecular therapies for IDD.

Nucleus pulposus cells (NPCs), the primary functional cells of the intervertebral disc, are essential for maintaining disc structure and function. Studies have shown that NPCs apoptosis, extracellular matrix (ECM) degradation, and abnormal pro‐inflammatory cytokine expression are key contributors to IDD [4]. Exposure to interleukin‐1β (IL‐1β) significantly increases NPCs apoptosis and ECM degradation, underscoring the role of inflammation in disc degeneration [5]. Bacterial lipopolysaccharides (LPS) are commonly used to simulate the inflammatory pathological microenvironment of intervertebral disc degeneration in vitro. Although LPS mainly induces acute cellular inflammatory injury and cannot fully replicate the chronic progressive process of IDD, a large number of studies have confirmed that this model can stably and reproducibly activate TLR4‐mediated signaling, significantly induce the secretion of pro‐inflammatory cytokines (TNF‐α, IL‐1β, IL‐6), promote nucleus pulposus cell apoptosis and extracellular matrix degradation, which are key pathological features of IDD [6, 7, 8, 9]. Therefore, the LPS‐induced NPC injury model is still a classic and reliable in vitro tool for preliminary screening of therapeutic targets and exploring inflammation‐related molecular mechanisms in IDD research.

Thrombospondin‐4 (THBS4), a member of the thrombospondin (THBS) family, is a calcium‐binding ECM protein predominantly expressed in the heart and other muscles [10, 11]. It plays a role in cell adhesion, tissue remodeling, nerve regeneration, and angiogenesis [12, 13]. Previous studies have shown that THBS4 is upregulated in various pathological conditions, including cardiac remodeling, atherosclerosis, and cancer and contributes to ECM stability by regulating collagen expression [14, 15]. THBS4 is a neuronally expressed ECM protein that promotes neurite outgrowth and is enriched in synapse‐rich regions [16]. Recently, elevated THBS4 expression has been observed in hepatocellular carcinoma tissues, indicating its potential role in disease progression [17]. Moreover, evidence suggests that THBS4 can activate the PI3K/AKT pathway via integrin β1, thereby promoting cell proliferation and inhibiting apoptosis [18]. Recent bioinformatics analyses have identified aberrant expression of THBS4 in disc degeneration [19]. However, expression patterns and potential mechanistic roles of THBS4 in IDD remain poorly understood and require further investigation.

The PI3K/AKT pathway is a key regulator of cell survival, inflammation, and homeostasis [20, 21]. Activation mitigates LPS‐induced apoptosis and inflammation [22, 23], and phosphorylation of the PI3K/AKT pathway can mitigate IDD by reducing ECM degradation and apoptosis in NPCs [24]. Furthermore, melatonin can inhibit high glucose‐induced excessive autophagy and apoptosis in NPCs by activating the PI3K/AKT pathway [25]. These findings suggest that the PI3K/AKT pathway plays a significant role in IDD progression. However, whether THBS4 exerts protective effects against LPS‐induced apoptosis and inflammation in NPCs through the PI3K/AKT pathway requires further investigation.

This study aimed to evaluate the expression and biological functions of THBS4 in LPS‐induced apoptosis and inflammation in NPCs. Furthermore, we investigated whether THBS4 exerted protective effects in IDD through the PI3K/AKT pathway, thereby providing experimental evidence to elucidate the regulatory mechanisms of THBS4 in IDD.

## 2 Materials and Methods

### 2.1 Bioinformatic Analysis

The Genecards (https://www.genecards.org/) were used to enter “intervertebral disc degeneration”, and the genes related to IDD in the databases were exported. Besides, the differential genes in IDD were obtained from the GSE186542 data set through analysis using GEO2R, an online tool integrated within the GEO database (https://www.ncbi.nlm.nih.gov/gds/). The intersecting genes were obtained by plotting the Venn diagram using an online tool (https://bioinfogp.cnb.csic.es/tools/venny/). KEGG pathway analysis was subsequently conducted by importing the intersecting genes into the KOBAS tool URL (http://bioinfo.org/kobas).

### 2.2 Cell Culture and Treatment

Human nucleus pulposus cells (NPCs) were purchased from ScienCell Research Laboratories (Carlsbad, CA, USA) and cultured according to the manufacturer's instructions. The cells were maintained in Dulbecco's modified eagle medium (DMEM)/F12 medium supplemented with 10% fetal bovine serum (FBS; Gibco, Grand Island, NY, USA) and 1% penicillin‐streptomycin (Gibco, Grand Island, NY, USA) at 37°C in a humidified incubator with 5% CO_2_. Passages 3–6 were used for all experiments, and the cells were subjected to treatment until they reached approximately 80% confluence.

To mimic the inflammatory microenvironment associated with IDD, NPCs were treated with 10 ng/mL LPS (Sigma‐Aldrich, St. Louis, MO, USA) for 24 h under serum‐free conditions. This dose and duration were strictly selected based on previously published studies that have well established and validated this condition for LPS‐induced inflammatory injury in human nucleus pulposus cells [26, 27].

To further assess the involvement of the PI3K/AKT pathway in THBS4‐mediated effects, we established rescue experiment groups: LPS + OE‐THBS4 (THBS4 overexpression under LPS stimulation) and LPS + OE‐THBS4 + LY294002 (THBS4 overexpression followed by PI3K/AKT pathway inhibition). For the pathway inhibition group, cells were pretreated with the PI3K inhibitor LY294002 (20 μM, Selleck Chemicals, Houston, TX, USA) for 1 h prior to LPS exposure. All inhibitors were dissolved in dimethyl sulfoxide (DMSO), and the corresponding solvent control groups were included in all assays.

### 2.3 Cell Transfection

The overexpression plasmid (OE‐THBS4) and a negative control vector (OE‐NC) were constructed and synthesized using GenScript (Nanjing, China). Cell transfection was performed using the Lipofectamine 3000 reagent (Invitrogen, Carlsbad, CA, USA) according to the manufacturer's protocol. Briefly, cells were seeded in 6‐well plates and transfected when they reached 70%–80% confluency. Plasmids and transfection reagents were diluted in Opti‐MEM to prepare the transfection complexes, which were then added to the cell culture medium.

### 2.4 RT‐qPCR

Total RNA was extracted from cells using TRIzol Total RNA Extraction Reagent (ELK Biotechnology, Wuhan, China). Reverse transcription was performed using the EntiLink 1st Strand cDNA Synthesis Kit (ELK Biotechnology, Wuhan, China) according to the manufacturer's instructions. The qPCR was conducted on a QuantStudio 6 Flex Real‐Time PCR System (Life Technologies, Gaithersburg, MD, USA) using EnTurbo SYBR Green PCR SuperMix (ELK Biotechnology, Wuhan, China). Each sample was run in triplicate, and ACTIN was used as an internal control. Relative gene expression was calculated using the 2^−ΔΔCt^ method. All primers were synthesized using GenScript (Nanjing, China), and their sequences are listed in Table 1.

**Table 1:** The RT‐qPCR primer sequences.

Name	Sequence(5′‐3′)	
ACTIN	Sense	GTCCACCGCAAATGCTTCTA
	Antisense	TGCTGTCACCTTCACCGTTC
THBS4	Sense	ATGTTTCCGAGGTGTCCAATG
	Antisense	GAAGCCAGGAGACAAATTTATGC

### 2.5 Western Blot Assay

Cells were lysed using radio immunoprecipitation assay buffer (ASPEN, Wuhan, China) supplemented with 1% protease and phosphatase inhibitors. Protein concentrations were determined using a bicinchoninic acid assay kit (ASPEN). Equal amounts of protein were separated by sodium dodecyl sulfate‐polyacrylamide gel electrophoresis and transferred onto polyvinylidene fluoride (PVDF) membranes (Millipore, Billerica, MA, USA). Membranes were blocked with 5% nonfat milk at room temperature for 1 h and then incubated overnight at 4°C with primary antibodies against THBS4 (sc‐390734, 1: 500, Source: Mouse, Santa Cruz Biotechnology, Santa Cruz, CA, USA), B‐cell lymphoma‐2 (Bcl‐2, #3498, 1: 1000, Source: Rabbit, Cell Signaling Technology, Danvers, MA, USA), Bcl2‐associated X (Bax, #2772, 1: 2000, Source: Rabbit, Cell Signaling Technology), Aggrecan (ab3778, 1: 500, Source: Mouse, Abcam, Cambridge, MA, USA), Collagen type II (ab307674, 1: 500, Source: Mouse, Abcam), p‐PI3K (AF3241, 1: 500, Source: Rabbit, affbiotech, Changzhou, China), PI3K (#4292, 1: 2000, Source: Rabbit, Cell Signaling Technology), p‐AKT (#4060, 1: 1000, Source: Rabbit, Cell Signaling Technology), AKT (#9272, 1: 3000, Source: Rabbit, Cell Signaling Technology), and β‐actin (TDY051, 1: 10000, Source: Rabbit, Beijing TDY Biotech co. Ltd., Beijing, China). The following day, the membranes were washed three times with tris buffered saline tween (TBST) and incubated with horseradish peroxidase‐conjugated secondary antibodies (AS1107/AS1106, 1: 10000, ASPEN) at room temperature for 1 h. Protein signals were visualized using an enhanced chemiluminescence (ECL) detection kit (ASPEN) and imaged with a ChemiDoc MP imaging system (Bio‐Rad, Hercules, CA, USA). β‐actin was used as an internal control, and band intensities were quantified using ImageJ software.

### 2.6 EdU Cell Proliferation Assay

Cell proliferation was assessed using an EdU‐488 cell proliferation detection kit (Servicebio, Wuhan, China). Briefly, NPCs were seeded into 24‐well plates and treated according to the experimental groups once cell confluence reached 70%–80%. Cells were then incubated with 10 μM EdU working solution for 2 h. After incubation, cells were gently washed twice with phosphate‐buffered saline (PBS), fixed in 4% paraformaldehyde for 30 min, and permeabilized with 0.5% Triton X‐100 for 20 min. The staining solution was prepared according to the manufacturer's instructions and applied for 30 min at 37°C in the dark. Nuclei were counterstained with Hoechst 33342. Fluorescence images were captured using a fluorescence microscope (Olympus, Tokyo, Japan). ImageJ software was used to quantify the proportion of EdU‐positive cells, thereby evaluating the proliferative activity of each group.

### 2.7 Flow Cytometry Analysis of Apoptosis

Cell apoptosis was performed using an Annexin V‐FITC apoptosis detection kit (BD, San Jose, CA, USA). Briefly, treated NPCs were harvested and washed twice with PBS. The cells were then resuspended in 100 μL of 1× binding buffer, followed by the addition of 5 μL Annexin V‐FITC and 5 μL propidium iodide (PI). After gentle mixing, cells were incubated in the dark at room temperature for 15 min. Following incubation, the cells were washed twice with 1× binding buffer and resuspended in 500 μL of 1× binding buffer. The cell suspension was then filtered and transferred to a flow cytometry tube. Apoptotic cells were analyzed within 1 h using a CytoFLEX flow cytometer (Beckman Coulter, Miami, FL, USA), and data were processed using FlowJo software. The gating strategy for the flow cytometry analysis is shown in the Supporting Information S1.

### 2.8 Enzyme‐Linked Immunosorbent Assay (ELISA)

To evaluate the secretion of inflammatory cytokines, culture supernatants from each group were collected and centrifuged to remove debris. Human ELISA kits for TNF‐α, IL‐1β, IL‐6, and IL‐10 (Elabscience, Wuhan, China) were used according to the manufacturer's instructions. Briefly, the standards and samples were added to 96‐well plates pre‐coated with specific antibodies. After incubation and washing, detection antibodies, enzyme conjugates, and substrate solutions were added sequentially. The reaction was terminated, and absorbance was measured at 450 nm using a microplate reader (BioTek, USA). Cytokine concentrations in the supernatants were calculated based on standard curves.

### 2.9 Statistical Analyses

All experimental data are presented as the mean ± standard deviation (SD) from at least three independent experiments. Statistical analyses were performed using GraphPad Prism software (version 9.0; GraphPad Software, USA). Comparisons between two groups were conducted using an unpaired two‐tailed Student's *t*‐test, whereas comparisons among multiple groups were performed using one‐way analysis of variance (ANOVA) followed by Tukey's test. Statistical significance was set at *p* < 0.05. significant.

## 3 Results

### 3.1 THBS4 Is Downregulated in LPS‐Induced NPCs

**Figure 1:**
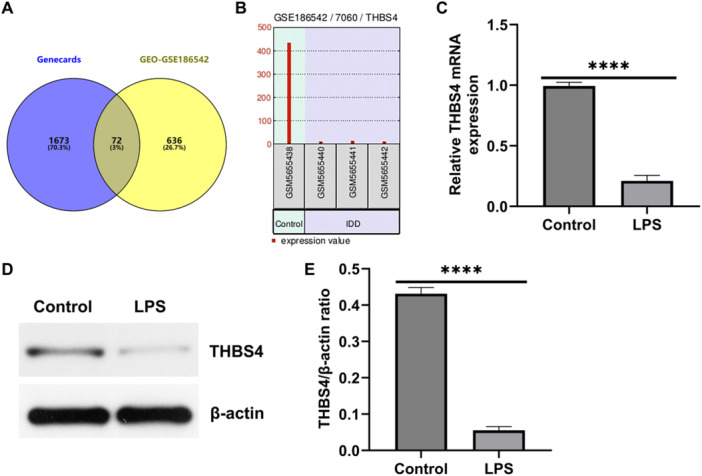
Downregulation of THBS4 in LPS‐treated NPCs. (A and B) Venn diagram of DEGs from GES186542 and genes associated with IDD in the Genecards database, identified THBS4 as significantly dysregulated in IDD; (C) RT‐qPCR analysis was used to measure the THBS4 mRNA levels in NPCs following LPS treatment; (D and E) Western blot analysis confirmed the THBS4 protein expression in the LPS‐treated group. Data are presented as mean ± SD. *N* = 3, biological replicate. ****p < 0.0001.

To explore the expression pattern and potential role of THBS4 in IDD, differential expression analysis was first performed using the IDD‐related transcriptomic dataset GSE186542 from the GEO database. The results were compared with the IDD‐associated genes retrieved from the GeneCards database using the keyword “intervertebral disc degeneration”. A total of 72 differentially expressed genes associated with IDD were identified (Supporting Information S2), among which THBS4 showed significant differential expression (Figure 1A,B), suggesting its potential involvement in the pathogenesis of IDD. To validate these bioinformatics findings, an in vitro inflammatory model was established by treating human NPCs with 10 ng/mL LPS for 24 h. RT‐qPCR revealed that LPS stimulation significantly suppressed the mRNA levels of THBS4 compared to the control group (Figure 1C). Consistent with this, western blot analysis confirmed the marked downregulation of THBS4 protein levels following LPS treatment (Figure 1D,E). These results indicated that THBS4 expression was significantly decreased in LPS‐induced NPCs, suggesting a potential regulatory role in inflammation‐mediated pathological processes relevant to IDD.

### 3.2 THBS4 Regulates LPS‐Induced Proliferation and Apoptosis in NPCs

**Figure 2:**
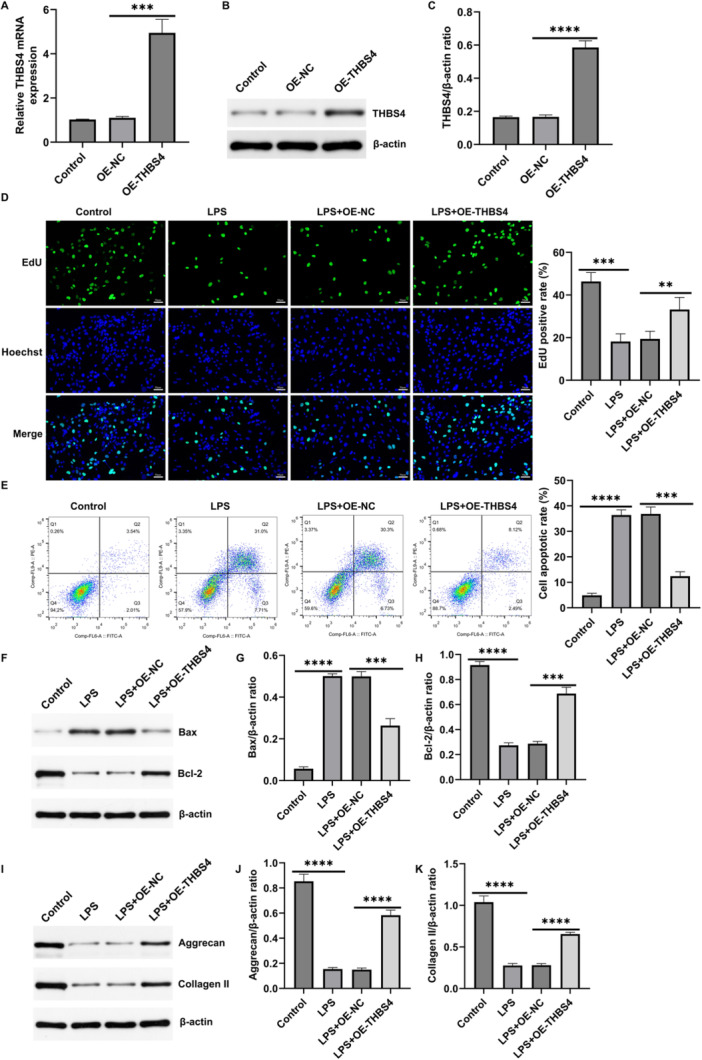
THBS4 regulates proliferation and apoptosis in LPS‐treated NPCs. (A–C) RT‐qPCR and western blot analysis of THBS4 expression levels; (D) EdU assesses cell proliferation (scale bar = 50 μm; magnification: 200×); (E) Flow cytometry analysis was performed to analyze cell apoptosis; (F–H) Western blot analysis of Bax and Bcl‐2 expression; (I–K) Western blot analysis of ECM‐related proteins aggrecan and collagen type II expression. Data are presented as mean ± SD. *N* = 3, biological replicate. **p < 0.01, ***p < 0.001, ****p < 0.0001.

To investigate the role of THBS4 in LPS‐induced injury in NPCs, THBS4 was overexpressed in an LPS‐induced NPCs model. After 48 h of transfection, RT‐qPCR and western blotting confirmed successful overexpression of THBS4 at both mRNA and protein levels (Figure 2A–C). EdU assays demonstrated that LPS treatment markedly suppressed NPCs proliferation, as indicated by a reduced proportion of EdU‐positive cells. In contrast, overexpression of THBS4 significantly restored the proliferative capacity (Figure 2D). Flow cytometry analysis revealed that LPS significantly increased apoptosis, whereas THBS4 overexpression markedly reduced apoptosis (Figure 2E). Western blot analysis consistently showed that LPS treatment upregulated Bax and downregulated Bcl‐2, whereas THBS4 overexpression reversed these effects, decreasing Bax and increasing Bcl‐2 expression (Figure 2F–H).

To assess the impact of THBS4 on the maintenance of the NPC phenotype, expression levels of the ECM proteins Aggrecan and Collagen type II were also evaluated. LPS stimulation significantly suppressed expression of both ECM markers, whereas THBS4 overexpression effectively restored their expression levels (Figure 2I–K).

These findings suggest that overexpression of THBS4 alleviates LPS‐induced inhibition of proliferation and enhancement of apoptosis in NPCs and promotes the recovery of ECM synthesis and cellular phenotype, indicating a potential protective role of THBS4 in the inflammatory damage associated with IDD.

### 3.3 THBS4 Modulates the Expression of Inflammatory Cytokines in LPS‐Stimulated NPCs

**Figure 3:**
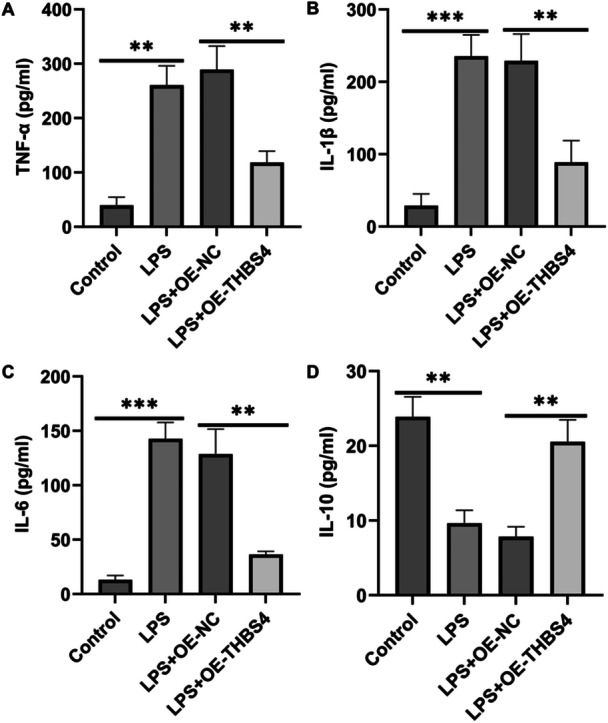
THBS4 regulates the secretion of inflammatory cytokines in LPS‐stimulated NPCs. (A–D) ELISA was used to assess the secretion of TNF‐α, IL‐1β, IL‐6, and IL‐10 in NPCs. Data are presented as mean ± SD. *N* = 3, biological replicate. **p < 0.01, ***p < 0.001.

To evaluate the effect of THBS4 on the inflammatory response of NPCs induced by LPS, ELISA was performed to quantify the levels of inflammatory cytokines TNF‐α, IL‐1β, IL‐6, and IL‐10 in culture supernatants. LPS stimulation significantly increased the secretion of TNF‐α, IL‐1β, and IL‐6, while markedly reducing the release of cytokine IL‐10 (Figure 3A–D). In the overexpression of THBS4 group, the levels of TNF‐α, IL‐1β, and IL‐6 were significantly decreased, whereas IL‐10 expression was significantly elevated compared to the LPS group (Figure 3A–D). These findings suggested that THBS4 exerts a prominent anti‐inflammatory effect on LPS‐induced inflammation in NPCs.

### 3.4 THBS4 Alleviates LPS‐Induced Damage in NPCs via Activation of the PI3K/AKT Pathway

**Figure 4:**
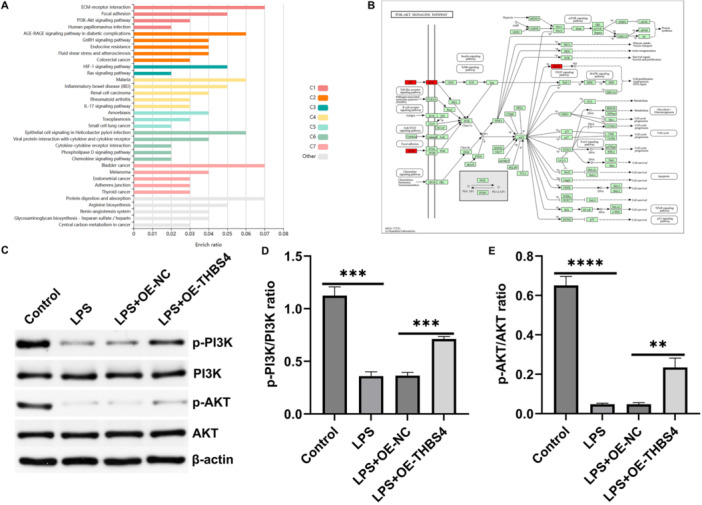
THBS4 alleviates LPS‐induced damage in NPCs by activating the PI3K/AKT signaling pathway. (A and B) KEGG pathway enrichment analysis of 72 IDD‐related differentially expressed genes revealed significant enrichment of the PI3K‐AKT signaling pathway, with THBS4 also mapped to this pathway; (C) Western blot analysis of PI3K, AKT, p‐PI3K, and p‐AKT in NPCs. (D) p‐PI3K/PI3K ratio. (E) p‐AKT/AKT ratio. Data are presented as mean ± SD. *N* = 3, biological replicate. **p < 0.01, ***p < 0.001, ****p < 0.0001.

To further explore the potential mechanism of THBS4 in IDD, KEGG pathway enrichment analysis was performed on the 72 IDD‐related differentially expressed genes identified earlier. The top 30 significantly enriched pathways included several inflammation‐ and apoptosis‐associated pathways such as PI3K‐AKT and NF‐κB pathways (Figure 4A). Notably, the PI3K/AKT pathway was significantly enriched and THBS4 was mapped within this pathway (Figure 4B, Supporting Information S3), suggesting that THBS4 may exert its biological functions by regulating PI3K/AKT signaling.

To validate this hypothesis, we examined the expression and phosphorylation of key proteins involved in the PI3K/AKT pathway. Western blotting revealed that LPS treatment significantly suppressed the phosphorylation of PI3K and AKT, with significantly reduced p‐PI3K/PI3K and p‐AKT/AKT ratios compared to those in the control group (Figure 4B–D). In contrast, THBS4 overexpression led to a significant increase in p‐PI3K and p‐AKT levels, effectively restoring the phosphorylation ratio (Figure 4B–D), indicating that THBS4 activates the PI3K/AKT pathway. These findings suggest that THBS4 exerts protective effects against LPS‐induced inflammatory responses, apoptosis, and functional impairment in NPCs in association with the activation of the PI3K/AKT pathway, with the specific regulatory mechanism to be further elucidated.

### 3.5 THBS4 Mediates LPS‐Induced Proliferation, Apoptosis, and Inflammation in NPCs via the PI3K/AKT Pathway: Validation by LY294002 Rescue Experiments

**Figure 5:**
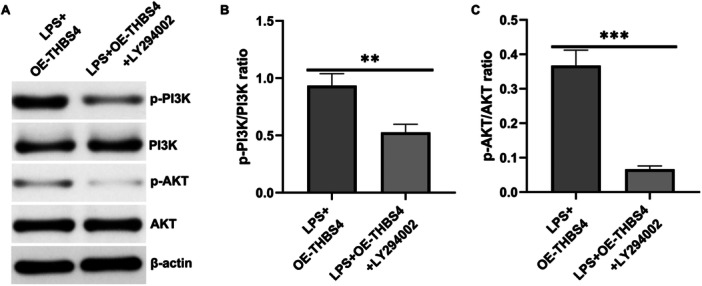
LY294002 suppressed PI3K/AKT signaling pathway in LPS‐induced NPCs. (A) Western blot analysis of PI3K, AKT, p‐PI3K, and p‐AKT in NPCs. (B) p‐PI3K/PI3K ratio. (C) p‐AKT/AKT ratio. Data are presented as mean ± SD. *N* = 3, biological replicate. **p < 0.01, ***p < 0.001.

To clarify the function of the PI3K/AKT pathway in the protective effects mediated by THBS4, we performed LY294002 rescue experiments in the LPS‐induced NPC model with THBS4 overexpression, and set up LPS + OE‐THBS4 and LPS + OE‐THBS4 + LY294002 groups to assess the impact of pathway inhibition on cellular functions. Signaling pathway analysis indicated that THBS4 overexpression markedly increased the phosphorylation of PI3K and AKT. LY294002 effectively inhibited the phosphorylation of PI3K and AKT (Figure 5A–C).

**Figure 6:**
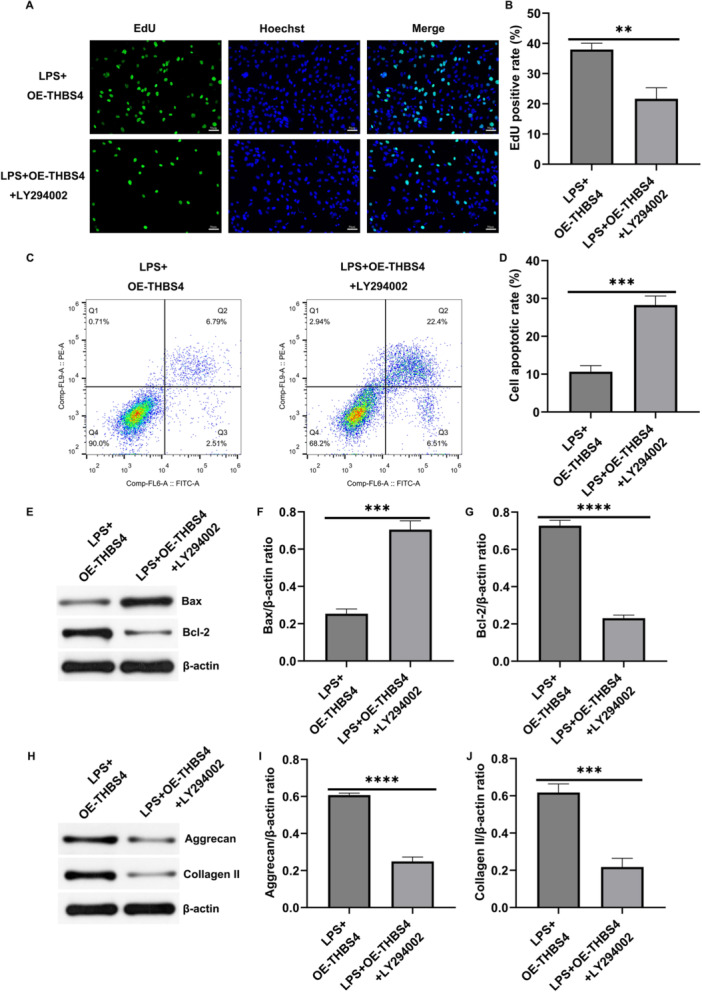
THBS4 regulates LPS‐induced proliferation and apoptosis in NPCs via the PI3K/AKT signaling pathway. (A and B) EdU assay determined NPC proliferation (scale bar = 50 μm; magnification: 200×); (C and D) Cell apoptosis was detected by flow cytometry analysis; (E–G) Western blot analysis of apoptosis‐related proteins including Bax and Bcl‐2; (H–J) Western blot analysis of extracellular matrix proteins including aggrecan and collagen type II. Data are presented as mean ± SD. *N* = 3, biological replicate. **p < 0.01, ***p < 0.001, ****p < 0.0001.

The EdU assay revealed that overexpression of THBS4 significantly enhanced NPCs proliferation, whereas LY294002 treatment markedly attenuated this proliferative effect (Figure 6A,B). Flow cytometry further demonstrated that THBS4 overexpression significantly suppressed LPS‐induced apoptosis, which was reversed by PI3K pathway inhibition (Figure 6C,D). Western blotting analysis revealed that THBS4 overexpression reduced Bax levels, increased Bcl‐2 expression, and decreased the Bax/Bcl‐2 ratio. In contrast, LY294002 treatment upregulated Bax expression and downregulated Bcl‐2 expression (Figure 6E–G).

**Figure 7:**
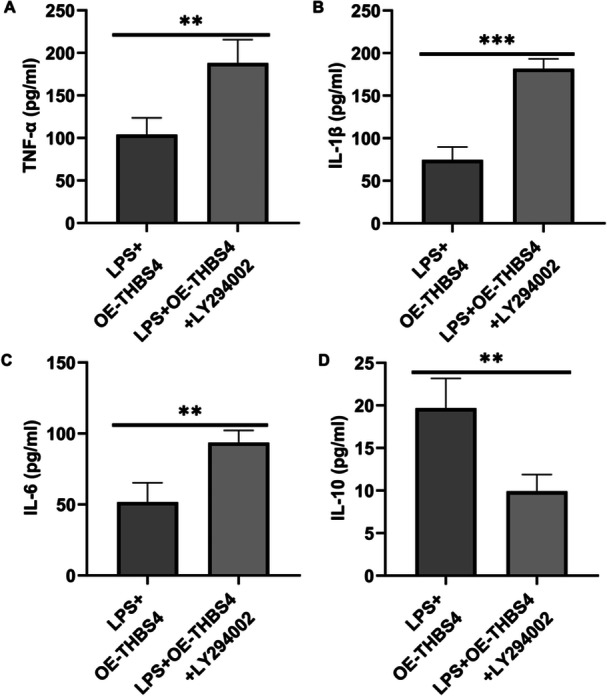
THBS4 regulates LPS‐induced inflammation in NPCs via the PI3K/AKT signaling pathway. (A–D) ELISA analysis of the secretion of TNF‐α, IL‐1β, IL‐6, and IL‐10 in NPCs. Data are presented as mean ± SD. *N* = 3, biological replicate. **p < 0.01, ***p < 0.001.

Regarding matrix phenotype maintenance, THBS4 overexpression significantly upregulated Aggrecan and Collagen type II expression, which was suppressed by LY294002 (Figure 6H–J). Figure 7A–D revealed that overexpression of THBS4 significantly inhibited the secretion of TNF‐α, IL‐1β, and IL‐6, while promoting IL‐10 expression. These anti‐inflammatory effects were reversed by PI3K inhibition (Figure 7A–D).

These findings indicate that THBS4 exerts multiple protective effects in NPCs, including anti‐apoptotic, anti‐inflammatory, and matrix‐preserving actions, which are dependent on the activation of the PI3K/AKT signaling pathway; the precise upstream molecular events underlying this pathway activation remain to be explored.

## 4 Discussion

Intervertebral disc degeneration is a leading cause of chronic low back pain and is characterized by a complex pathogenesis involving apoptosis, inflammatory responses, and an imbalance in ECM metabolism [28, 29]. Increasing evidence has highlighted the central role of the inflammatory microenvironment in IDD [30]. Among these processes, NPC dysfunction in response to inflammation is considered a pivotal factor driving disc degeneration [31]. Therefore, identifying novel molecular targets that regulate NPC injury is of great significance for early intervention and treatment of IDD.

This study demonstrated that THBS4 expression is significantly suppressed following LPS stimulation, a cellular model of IDD, suggesting its potential involvement in inflammation‐mediated cellular injury during IDD progression. THBS4, a calcium‐binding ECM glycoprotein, has been implicated in the repair of cardiac, neural, and tendon tissues [11, 32, 33]. Studies have shown that THBS4 deficiency impairs neuronal migration in mice [34]. Moreover, THBS4 stimulation activates pathways that regulate the proliferation, migration, and inflammation of human primary keratinocytes [35]. However, the role in IDD remains unexplored and has not yet been investigated.

Multi‐omics analyses have identified THBS4 as a central hub gene in degenerative intervertebral disc tissues, co‐enriched with ECM‐related genes such as COL6A2 and COL9A3, suggesting a potential regulatory role in the pathogenesis of IDD [36]. This study further confirmed the protective role of THBS4 in IDD. LPS‐ induced NP cell injury has been widely used for IDD study in vitro [37, 38]. Our results demonstrated that overexpression of THBS4 significantly alleviated LPS‐induced injury in NPCs, as evidenced by enhanced cell proliferation, reduced apoptosis, upregulated expression of cartilage matrix proteins (Aggrecan and Collagen type II), decreased secretion of pro‐ inflammatory cytokines (TNF‐α, IL‐1β, and IL‐6), and increased expression of the anti‐inflammatory cytokine IL‐10. Mechanistically, THBS4 activated the PI3K/AKT signaling pathway by promoting the phosphorylation of PI3K and AKT. Notably, the protective effects of THBS4 were markedly reversed by the PI3K inhibitor LY294002, indicating that THBS4 exerts anti‐apoptotic and anti‐inflammatory effects primarily through the activation of the PI3K/AKT pathway. Notably, previous studies have demonstrated that THBS4 interacts with integrin β1 (ITGB1) and regulates the progression of hepatocellular carcinoma via the FAK/PI3K/AKT signaling pathway [18]. It is therefore speculated that THBS4 may activate the PI3K/AKT pathway in NPCs through the integrin‐FAK axis, which is a potential upstream regulatory mechanism to be verified in subsequent research.

In the present study, we focused on the PI3K/AKT pathway for in‐depth mechanistic validation, and this experimental design was based on our preliminary KEGG pathway enrichment analysis. Our KEGG analysis identified multiple IDD‐associated signaling pathways, including PI3K/AKT and NF‐κB, all of which are involved in IDD pathogenesis. We focused on the PI3K/AKT pathway for experimental verification due to two key considerations: this pathway is a well‐established core regulator of NPC proliferation, apoptosis, inflammation, and extracellular matrix metabolism, and existing studies have confirmed a direct molecular association between THBS4 and the PI3K/AKT pathway. Other enriched pathways were not examined in the present study, as our work was designed to clarify the primary mechanistic role of THBS4 in LPS‐induced NPC injury, with inherent scope limitations of a single study. Given the potential crosstalk between these pathways in inflammatory responses, the interaction of THBS4 with other KEGG‐enriched pathways (e.g., NF‐κB) in IDD will be investigated in our subsequent research.

The critical role of the PI3K/AKT pathway in IDD is also well supported by a large body of previous studies, which further validate the rationality of our research focus. Previous studies have consistently demonstrated that the PI3K/AKT pathway plays a pivotal role in maintaining intervertebral disc cell homeostasis [22, 39, 40]. Wu et al. reported that TSG‐6 inhibits inflammatory responses, ECM degradation, and expression of pain‐related molecules in human NPCs by activating the PI3K/AKT pathway [41]. Moreover, several studies have shown that activation of the PI3K/AKT pathway effectively mitigates NP cell inflammatory injury. For example, maltol inhibited NP cell injury through the suppression of the PI3K/AKT/NF‐κB signaling pathway [42]. Similarly, Astragaloside IV alleviates IL‐1β‐induced degeneration of human NPCs by modulating the PI3K/AKT signaling pathway [43]. In addition, pharmacological agents such as Kukoamine A have been shown to mitigate LPS‐induced apoptosis, ECM degradation, and inflammation in NPCs by activating the PI3K/AKT pathway [44]. Collectively, these findings suggest that activation of the PI3K/AKT pathway not only suppresses inflammation and apoptosis, but also promotes ECM synthesis, highlighting its therapeutic potential in slowing the progression of IDD.

This study provides the first systematic evidence that THBS4 confers protective effects in an IDD model by activating the PI3K/AKT signaling pathway, highlighting its potential as a key anti‐inflammatory and anti‐apoptotic regulator. These findings offer a theoretical basis for advancing our understanding of the pathogenesis of IDD and developing targeted therapeutic strategies. Despite the innovative findings of this study, it has several limitations. First, the LPS‐induced in vitro inflammatory model mainly simulates acute inflammatory injury rather than the full chronic degenerative process of IDD, and in vitro models cannot fully replicate the complex in vivo pathological microenvironment; therefore, further in vivo studies using chronic IDD animal models are needed to fully validate our conclusions. Second, this study only verified the activation of the core PI3K/AKT pathway by THBS4 but did not detect the upstream (e.g., FAK, integrins) and downstream (e.g., GSK3β, mTOR) key markers of the pathway, thus the precise molecular mechanism by which THBS4 regulates the PI3K/AKT pathway in NPCs cannot be fully ascertained; in addition, the basal effects of THBS4 were not further verified in the experimental design, and future studies will detect these key signaling molecules to clarify the complete regulatory axis of THBS4‐mediated PI3K/AKT activation and supplement the verification of basal THBS4 effects to further improve the mechanistic inference of this study. Third, in the rescue experiments of this study, we did not establish an LPS + OE‐NC group; the inclusion of this group would have made the analysis of LY294002's reversal effect on OE‐THBS4 in LPS‐induced NPCs more intuitive. Fourth, the current evidence for THBS4 downregulation in IDD is limited to bioinformatic analysis of IDD‐related transcriptomic datasets and in vitro LPS‐induced NPC models, lacking validation from clinical IDD tissue samples (e.g., via qPCR, IHC) and in vivo IDD animal models. Future research will focus on verifying the expression pattern of THBS4 in clinical IDD samples and in vivo models to further confirm its regulatory role in IDD progression. Additionally, future studies will explore the crosstalk between THBS4 and other KEGG‐enriched pathways (e.g., NF‐κB) in IDD to fully elucidate its comprehensive regulatory mechanism.

In conclusion, this study is the first to demonstrate that THBS4 exerts protective effects by modulating LPS‐induced apoptosis and inflammation in NPCs through the activation of the PI3K/AKT signaling pathway. These findings identify THBS4 as a promising molecular target for therapeutic intervention in IDD and provide new insights into the pathomechanisms underlying IDD and its precise treatment.

## Author Contributions

**Yang Shu:** conceptualization, methodology, investigation, writing – original draft, writing – review and editing, formal analysis, project administration, data curation. **Chunhua Sun:** investigation, writing – original draft, methodology, visualization, formal analysis, data curation, supervision. **Shi Chen:** data curation, writing – review and editing.

## Funding

The authors have nothing to report.

## Ethics Statement

The authors have nothing to report.

## Consent

The authors have nothing to report.

## Conflicts of Interest

The authors declare no conflicts of interest.

## Supporting information

Supporting File 1

Supporting File 2

Supporting File 3

## Data Availability

The data that support the findings of this study are available from the corresponding author upon reasonable request.
