# Supplementary figures and images for: Thrombospondin‐4 Regulates Lipopolysaccharide‐Induced Apoptosis and Inflammation in Nucleus Pulposus Cells via the Phosphatidylinositol 3‐Kinase/Protein Kinase B Pathway
Source: Immun Inflamm Dis. 2026 Jul 6;14(7):e70484. doi: 10.1002/iid3.70484 (PMC13338625; doi:10.1002/iid3.70484)

**The gating strategy for the flow cytometry analysis:**


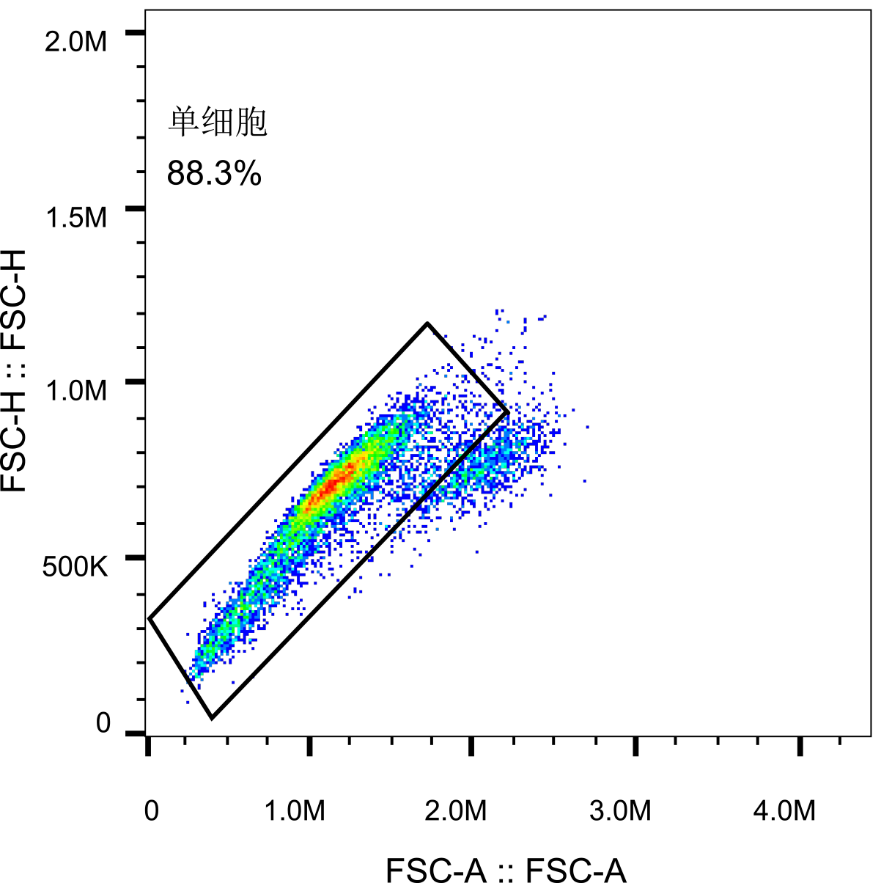


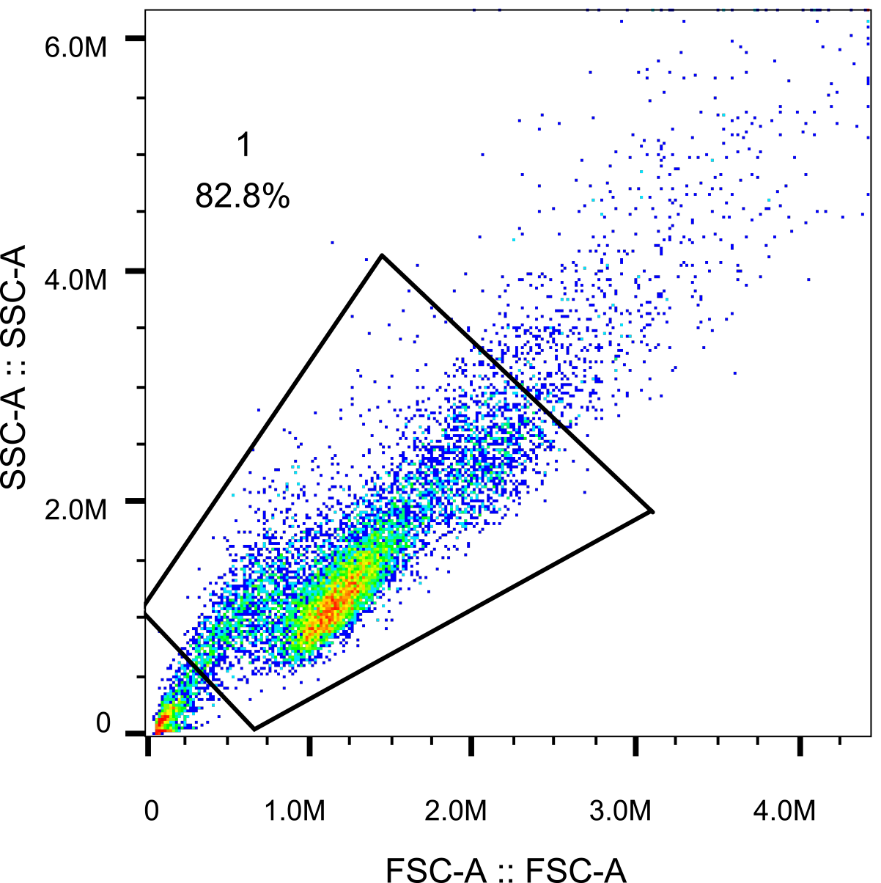

Supplement: Supplementary file 1 — Supporting File 1 [file IID3-14-e70484-s001.docx]
